# Thermoregulation and Performance of Dairy Cows Subjected to Different Evaporative Cooling Regimens, with or without Pepper Extract Supplementation

**DOI:** 10.3390/ani12223180

**Published:** 2022-11-17

**Authors:** Sidinei Peretti, Viviane Dalla Rosa, Maria Luísa Appendino Nunes Zotti, Alan Miranda Prestes, Patrícia Ferreira Ponciano Ferraz, Aleksandro Schafer da Silva, Claiton André Zotti

**Affiliations:** 1 Graduate Program in Animal Health and Production, University of the West of Santa Catarina (UNOESC), Rua Dirceu Giordani, n.696, Jardim Taruma, Santa Catarina, Xanxerê 89820-000, Brazil; 2 Department of Animal Science, State University of Santa Catarina (UDESC), St. Beloni Trombeta Zanin, 680E, Santa Catarina, Chapecó 89815-630, Brazil; 3 Department of Agricultural Engineering, Federal University of Lavras (UFLA), Lavras 37200-900, Brazil

**Keywords:** behavior, efficiency, heat stress, metabolic parameters, milk production

## Abstract

**Simple Summary:**

Heat stress affects the comfort, health, production, and fertility of dairy cows (*Bos taurus*). Among options to attenuate heat stress is the use of a cooling strategy combining wind and water, and another is incorporating feed ingredients or additives that promote body thermoregulation. The objective of this study was to evaluate whether there is an interaction between pepper extract supplementation and different evaporative cooling regimens on the performance, thermal comfort, and metabolism of lactating cows. The use of sprinklers for 30 s every 5 min resulted in greater milk yield and efficiency. Pepper extract reduced surface temperature and increased the time that cows spent at the feeding line. This study provides insights into the association of feed additives and cooling strategies to minimize heat stress and improve performance. Both of the tested evaporative cooling strategies produced remarkable results independent of the use of pepper extract.

**Abstract:**

The objective of this study was to evaluate whether there is an interaction between pepper extract (PE) supplementation and evaporative cooling regimens on the performance, thermal comfort, and metabolism of lactating cows. The experiment was designed as a double 4 × 4 Latin square using eight multiparous Holstein cows (days in milk 147 ± 43.8 days). Treatments were a 2 × 2 factorial arrangement; two evaporative cooling regimens [sprinkler cycles of 30 s on and 4.5 min off (R5) and 30 s on and 9.5 min off (R10)] were combined with no inclusion of pepper extract (CT) or 800 mg/cow daily of PE. The inclusion of PE allowed a greater reduction in the surface temperature of the coat, and the cows remained for longer periods in the feeding area. There was an interaction between the use of PE and the climate regimen for surface temperature, which was lower for PER5. Cows experiencing greater intermittency in the spraying cycles (R10) spent 35% more time at the feeding area. A significant interaction was observed for milk production, with greater production for CTR5 compared to the other treatments. The feed efficiency for R5 was higher than that for R10. The R5 treatment combined with PE reduced water intake. There was no significant difference in serum parameters other than gamma-glutamyl transferase, with an interaction between treatments and greater activity for PER10, and total protein and albumin, which had cooling effects that were higher for R10. The two factors tested interfered in different and dissociated ways. The evaporative cooling strategies were effective, and the R5 treatment resulted in higher feed efficiency and milk production. The inclusion of PE enhanced heat reduction in cows when they were subjected to a cooling regime.

## 1. Introduction

At a time of increasing global demand for food, there has been a tendency to increase the number of feedlots in Brazil and worldwide. These changes in the world scenario of production systems are necessary to meet the demand for animal protein, especially for meat and milk [1]. The strong pressure from entities linked to animal welfare means that facilities must be designed to meet the standards of animal comfort, welfare, and health, in addition to good management practices, with an emphasis on production system sustainability [2].

The southern region of Brazil accounts for a large share of national milk production, i.e., 33.5% of the total milk produced in the country [3]. In this region, there is a large thermal amplitude because it is located below the Tropic of Capricorn in a temperate zone, thus being subject to high temperatures [4]. High temperatures and high relative humidity are the two main elements responsible for causing heat stress in dairy cows [5]. Even during colder seasons, such as in spring, with the increase in temperature throughout the day, exceeding the lower effort range or thermoneutral zone, associated with increasingly productive animals with high metabolic activity, the adverse effects of heat stress require a recovery period for animals that can last up to 5 days [6].

The authors in [7] reported that heat stress affects dairy cattle, making it difficult to maintain adequate levels of herd comfort, health, production, and fertility, thus causing losses to dairy farms. It is estimated that this thermal stress is responsible for a 21% loss in milk production by Holstein cows in the southern region of Brazil [8]. In another study in the USA, there was a reduction of 0.56 L of milk per cow/day when the animals were subjected to a temperature range of 24 to 35 °C [9]. Thus, changes and adaptations in daily management are necessary to minimize the effects of heat stress on lactating cows [10].

The adoption of an evaporative cooling system, which consists of combining water spray with forced ventilation to dissipate heat from the body surface of animals, favors thermal comfort [5]. Studies using this type of cooling, called direct cooling, have reported lower surface and rectal temperatures [11,12], an 8% reduction in respiratory rate, and an increase in milk production of approximately 8.6% [13,14]. However, the use of water resources in animal production has been a point of environmental concern, requiring efficient management of such resources [15]. In light of the positive impact on animal welfare, the costs (water and energy) should be minimized; it is important to define an ideal cooling regime, considering improvements in thermal comfort, animal performance, and environmental responsibility.

In addition to the adoption of thermal conditioning methods, other strategies are needed to maximize the reduction in thermal stress, for example, dietary manipulation, with the inclusion of additives. Among additives, plant extracts such as capsaicin are promising because of their role in mechanisms that promote body thermoregulation [16], and their effect may be beneficial for animals prone to heat stress. As transient receptor potential vanilloid 1 (TRPV-1) ion channels are present in a large number of cells and tissues, the potential of capsaicinoids to stimulate or desensitize these receptors has been the target of research not only for the treatment of pain but also in relation to their role in thermoregulation, gastrointestinal function, and food intake regulation [13].

There are no studies in the literature that describe the interaction between spraying regimens and pepper extract (PE) supplementation with the goal of maximizing the efficiency of the use of natural resources and the productive efficiency of animals under thermal stress. In this study, high-production cows were subjected to two evaporative cooling regimens (sprinkler cycles of 30 s on and 4.5 min off or 30 s on and 9.5 min off), supplemented or not with PE. We evaluated the impact of different cooling and supplementation regimens using a low capsaicin dosage and different spray volumes per cycle, and our hypothesis was that a 50% reduction in the volume of water in the presence of PE supplementation would provide similar thermoregulatory and productive conditions. Thus, the objective of this study was to evaluate whether there is an interaction between PE supplementation and different evaporative cooling regimens on the productive performance, thermal comfort, and metabolism of lactating cows.

## 2. Materials and Methods

### 2.1. Design, Animals, and Diet

This experiment was conducted at a commercial farm located in the municipality of Xavantina, Santa Catarina. The experimental design was a duplicated 4 × 4 Latin square in a 2 × 2 factorial arrangement. Eight multiparous Holstein cows (days in milk 147 ± 43.8 days), with a mean body weight of 600 ± 52.6 kg and milk production of 35 ± 5.2 kg/day, were used in the experiment. Four experimental periods of 21 days each, with 14 days of adaptation and 7 days of sampling, were used. During the adaptation of the animals to the second period, 2 cows were replaced, 1 due to mastitis and the other due to allergic dermatitis.

The treatments consisted of 2 evaporative cooling regimens [sprinkler cycles of 30 s on and 4.5 min off (R5) and 30 s on and 9.5 min off (R10)] and the use [800 mg/cow daily (PE)] or not of PE. The PE contained 5 g/kg capsaicin (Capsin^®^; NutriQuest, Campinas, Brazil), and the dose use was followed according to the company recommendation.

The use of 2 evaporative cooling regimens was chosen, aiming for a 50% reduction in water volume from 10 to 5 min (R10 to R5). The sprinkler and ventilation systems were continuously automated, and the system was activated whenever the temperature exceeded 22.2 °C, as previously described by [15]. Under thermal conditions above 22.2 °C, the sprinkler system was intermittent (30 s on and 4.5 min off [R5] or 30 s on and 9.5 min off [R10]); however, ventilation was constant, regardless of the air temperature. The flow rate coefficient adopted was 4 ± 0.42 L/min. During the experimental period, there was 1 day when the system was not activated; the data for this day were excluded.

PE (800 mg/cow daily) was supplied to the animals and mixed with 100 g of ground soybean hull in the morning, ensuring its total consumption. Cows that did not receive PE were fed ground soybean hulls only. The basal diet (Table 1) was offered as a total mixed ration (TMR) twice daily (at 6:30 and 17:30), formulated in accordance with the nutritional recommendations of the [13] for cows in the middle third of lactation, with a mean body weight of 600 kg and milk production of 35 kg/day, containing 4% fat and 3.3% protein.

### 2.2. Facilities and Equipment

**Figure 1:**
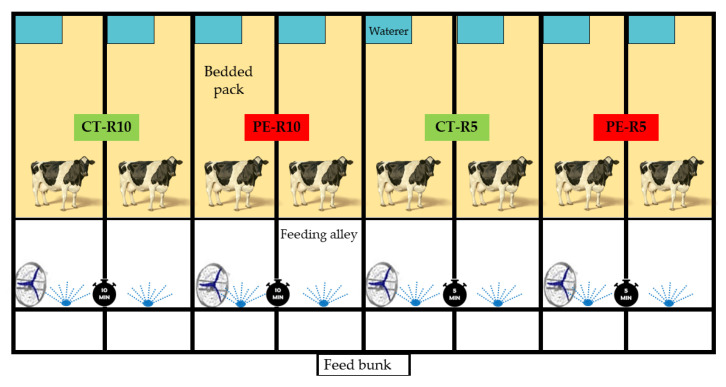
Schematic diagram of the trial: dairy cow barns with sprinkle and fan cooling systems.

The cows were housed in compost barn-type individual pens (16 m^2^) that included a feeding area and litter and had ad libitum access to water and total diet. The entire installation is 200 m^2^, with a ceiling height of 4 m, covered with fiber cement tiles, and it has an east–west solar orientation (Figure 1).

The cooling process consisted of spraying cycles and constant ventilation. The flow rate of each sprinkler was 4 L per minute. Water sprinklers were used on the feeder line (FloodJet^®^, Spraying Systems Co., Wheaton, IL, USA), which allowed wetting the back and sides of each animal. The spray nozzles were positioned 2 m above the floor, covering a radius of 2 m, with a 207 kPa water pressure regulator (30 psi, 2.1 kg/cm^2^). The fans were positioned at a height of 2.5 m from the floor and inclined at 30° over the feed line. Ventilators (diameter, 0.9 m) were spaced every 10 m and were equipped with a 1/4 hp motor providing a flow rate of 300 m^3^ h^−1^ at 495 rpm, with the capacity to produce air movement of up to 2.5 ms^−1^.

### 2.3. Animal Performance and Chemical and Physical Analyses of the Diet

**Figure 2:**
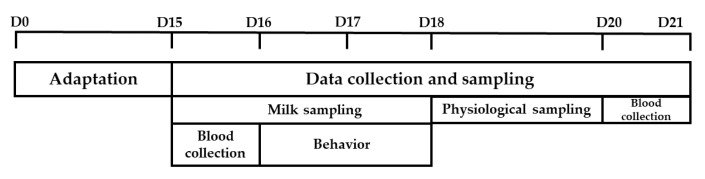
Schematic diagram of the experiment. Milk samples were collected every milking over 3 consecutive days (D15 to D18). Blood collection was performed on D15 and D21. Daily behavior was analyzed on D16 and D17. Physiological variables were collected from D18 to D20.

The supplied diet and leftovers were weighed and recorded daily during the 7 days of collection (Figure 2) to estimate dry matter intake. Samples of corn silage and total diet were collected twice a week for particle fractionation and dry matter analysis. Samples of TMR and leftovers were collected daily, mixed at the end of each collection period, and frozen until bromatological analysis.

Particle fractionation of the total diet and corn silage was performed in accordance with the Penn State particle separator methodology [18], and the selection index was calculated using the methodology described by [19]. Chemical analyses were performed in the bromatology laboratory on the campus of UNOESC Xanxerê. Dry matter (DM) (930.15), organic matter (OM) (942.05), ash (942.05), crude protein (CP) (954.01), and ether extract (EE) (Soxhlet) (920.39) were analyzed in accordance with the methodology described by [20]. Neutral detergent fiber (NDF) and acid detergent fiber (ADF) were analyzed using the methodology described by [21] with modifications suggested by [22]. Starch was analyzed as described by [23].

### 2.4. Characterization of the Thermal Environment

HOBO dataloggers (model U12), with an accuracy of ±0.35 °C for temperature and ±2.5% for relative humidity, were installed in each pen. They were coupled to the black globe in the external channel of each stall that recorded the dry bulb temperature (°C), relative humidity (%), and black globe temperature (°C) every 30 min for 7 consecutive days during each experimental period (Figure 2). The recorders were placed near the feeder line, 30 cm above the height of the animals, thus preventing them from being wetted by the sprinklers. These data were used to calculate the temperature and humidity index (THI) [24] using the following equations: THI = Tdb + 0.36 Tbu + 41.5, where Tbd refers to the dry bulb temperature (°C), and Tbu refers to the wet bulb temperature (°C).

A portable digital propeller anemometer (Incoterm^®^, Model 7607.01.0.00, Brasília, Brazil) was used to record the air velocity in each pen 3 times per day (8:00, 14:00, and 20:00). For this purpose, the equipment was positioned at the height of the animals.

The dry bulb temperature and relative air humidity were analyzed with respect to the mean values and amplitudes within 24 h in addition to the values at 8:00 to 20:00. Based on the results of a previous analysis, it was decided to present the thermal environment data for the facility as a whole, without differentiating between treatments.

### 2.5. Performance and Milk Composition

The cows were milked twice per day (4:00 and 16:00) in a herringbone milking parlor, consisting of equipment with electronic production records. The daily milk production (total production at each milking) was recorded over the 7 days of collection in each period (D15 to D21), and milk composition was recorded individually for 3 consecutive days (D15 to D17) in each experimental period.

Individual samples were collected, and the milk composition (fat, protein, and lactose content) and quality (somatic cell count) were determined in accordance with ISO 9622/IDF 141, calibrated using ISO 6731/IDF 021, IDF 001/ISO 1211, ISO 22662/IDF 198 and 8968-1/IDF 20-1, as established in IN 77 [25], as a reference method used by official laboratories.

The feed efficiency (FE) was calculated by dividing the daily milk production by the daily DM intake. Milk production corrected for fat and energy was calculated using the following equations: 4% FCM = milk production (kg/day) × [milk fat (%) × 0.15 + 0.4] and ECM = milk production (kg/day) × {[0.3887 × milk fat (%)] + [0.2356 × milk protein (%)] + [0.1653 × milk lactose (%)]}/3.1338 [26].

### 2.6. Serum Parameters

On the first (D15) and last days of collection (D21) of each experimental period, blood samples were collected from the coccygeal vein using Vacutainer tubes with EDTA and Serum separator tube to determine glucose, albumin, globulin, cholesterol, and urea levels. In the biochemical analyses, the serum levels of total protein (TP), glucose, albumin, urea, AST, and GGT were evaluated using a Bio-2000 Bioplus^®^ (São Paulo, Brazil) semiautomatic analyzer and Analisa^®^ (Belo Horizonte, Brazil) commercial kits. Globulin levels were determined by the difference between total protein and albumin.

### 2.7. Behavior

The behavior of the animals was recorded on D16 and D17 of each experimental period (Figure 2) using video cameras. The images were subsequently analyzed by a trained observer who recorded the behavior of the animals for a period of 48 continuous hours in each experimental period, following 2 different methodologies.

The presence of an animal in the feeding track was recorded every time within a period of 48 h, with the recording of the start and end times, which allowed obtaining the number of visits, partial time, and total time that the animals were exposed to the climate control system [15,27]. Specifically, the estimated number of visits to the feeding track was based on the presence of the animal in the track for more than 30 s, i.e., the spraying time of each cycle, which was also 30 s.

In addition, throughout the 48 h, the behavior of the animals was recorded every 30 min using instantaneous recording with a focal animal [28]. During milking (30 min duration), behavioral recording was not performed, totaling 46 recordings over the 48 h of observation, obtaining an estimate of how many minutes per hour the animals were in each behavioral category, i.e., posture, activity, and location.

The working ethogram used for the instantaneous recording independently considered posture, activity, and location. Posture was considered standing (animal standing or in transition from standing to lying down or lying down to standing) or lying down (animal lying down in lateral or sternal decubitus). Activity was considered leisure (animal stopped, without performing any activity), ruminating, in movement (animal moving in the litter area or in the feeding track), eating, drinking, and other (actions such as scratching, licking, social interaction, and other activities not described). Finally, location was considered in the eating area or in the pen. The behaviors were recorded by the instantaneous method.

Additionally, the latency time of water consumption after food was supplied was measured. For this purpose, the animals were continuously observed during the feeding of the diet, and after leaving the feeder line, the time (in minutes) was recorded from the end of eating to when the animal began to consume water in minutes.

Each cow received a collar (SCR Engineers Ltd., Netanya, Israel) that could measure rumination activity during the 7 days of collection in each period. The monitoring system automatically provided the data, which were exported to an Excel spreadsheet at the end of each experimental period.

### 2.8. Thermoregulatory Variables

The physiological variables indicative of thermoregulatory processes were measured for 3 consecutive days (D18, D19 and D20) at 8:00, 14:00, and 20:00. The first physiological response measured was the respiratory rate (RR), with the recording of the time spent to achieve 10 flank movements and the subsequent calculation of movements/min [15]. The rectal temperature (RT) was measured by a digital clinical thermometer in contact with the rectal mucosa. The surface temperature (ST) was obtained in the thorax (rib) region of the cows, always on the right side, using an infrared thermometer with a resolution of 0.2 °C, adjusted for an emissivity of 0.98 [29].

### 2.9. Statistical Analysis

All data were analyzed using the MIXED procedure in [30] for a 4 × 4 Latin double square design. The statistical model included the fixed effect of the air conditioning regimens (R5 or R10), the use (PE) or not (CT) of PE, and the interaction between them. The experimental period, pen, and cow within the pen were considered random effects. The LED was considered a covariate for the milk production and composition data. Except for physiological variables (CS matrix), the other variables were analyzed using a covariance matrix. Comparisons between treatments were performed using the Tukey test; *p* ≤ 0.05 was considered significant, and 0.05 ≥ *p* ≤ 0.10 was considered a trend.

## 3. Results

### 3.1. Environmental Conditions

The mean thermal conditions and oscillation values recorded throughout the experiment are presented in Table 2 for the meteorological variables and the environmental indices used to characterize the thermal environment. For air velocity, an average value of 2.5 ± 0.15 m/s was obtained.

### 3.2. Physiological Parameters

There was no significant difference between the RR treatments, and there was only a trend (*p* = 0.0748) toward a decrease in RR when the animals received PE (Table 3). The RR differed only as a function of the time of data collection (*p* < 0.0001), with a progressive reduction in the RR throughout the day. There was an interaction between the use of PE and the climate control regimen for ST, with a lower value for the PER5 treatment than for the other treatments, which did not differ from each other. As a function of time, ST was significantly lower at 14:00 and 20:00 than at 8:00 (*p* < 0.0001). For RT, there was no significant difference (*p* < 0.05) for either treatment or time of day.

### 3.3. Behavioral Parameters

There was an interaction effect between the use of PE and the cooling regimen in the mean time per visit to the feeding track and therefore in the exposure time to acclimatization per visit (Table 4). When the animals did not receive PE, the mean length of stay in the feeding lane per visit was higher for R5 than for R10. The same did not occur when animals received PE. That is, animals that received the CTR10 treatment had a shorter time per visit than did animals that received the CTR5 treatment, with a permanence of 35% less, with no difference among the other treatments. However, the total time in which the animals were exposed to air conditioning, the number of visits, and the latency were not significantly different (Table 4).

The behaviors of the animals were classified into posture, activity, and location (Table 5). There was a trend for PE to decrease the frequency of lying down (*p* = 0.0837) and increase the frequency of standing (*p* = 0.0666). In terms of leisure activities, i.e., ruminating, moving, eating, and drinking, there was a significant difference only for eating (*p* = 0.0375), with higher values when the animals consumed PE. The ruminating activity measured by a monitoring system or by behavioral observation did not differ significantly among the different treatments.

The location of the animals in the feeding area was higher for the animals that received PE (*p* = 0.0134), with no significant difference (*p* < 0.05) for the evaporative cooling regimen. In the litter area, the permanence time was lower for PER5 and PER10 and differed for CTR5 and CTR10 (*p* = 0.0192).

### 3.4. DM Intake and Milk Production and Composition

There was no significant interaction between the use of PE and the climate control regimens for DM intake. There was a significant increase in DM intake (*p* = 0.0205) when the animals were subjected to the R5 intermittent regimen compared to the R10 regimen (Table 6).

A significant interaction was observed for milk production, with higher production for CTR5 than for the others. Furthermore, the interaction between PE use and acclimatization regimens for ECM was significant, with higher production for CTR5 and lower production for CTR10; these results were not different from those of the treatments with PE (Table 6). However, the use of PE in the R10 treatment resulted in an increase of 2 and 4.4% compared to the control for milk production and ECM, respectively. The milk production corrected for 4% fat (FCM) increased for the R5 regime, with no effect of the addition of PE. The fat content did not differ between treatments, while the daily fat production tended to increase (*p* = 0.089) with the use of R5, with no effect for PE.

There was an interaction between the use of PE and the climate control regimen in milk protein content (*p* = 0.0028), showing a trend toward PE reducing this variable for R10, and conversely, CT increased this variable for R10. For protein yield (kg/day), there was a significant interaction between the use of PE and the climate control regime, with the CTR5 treatment resulting in higher production than that for other treatments. There was no difference in lactose content, with only a trend toward an interaction between treatments (*p* = 0.0917). Lactose production had an interaction effect, with higher production for the CTLR5 treatment than for the others. Likewise, the R5 climate regimen resulted in better feed efficiency only in the control treatment and did not differ when PE was used. The feed efficiency from ECM and FCM increased with a greater number of spray cycles (R5 to R10). There was no effect of the treatments on somatic cell count.

### 3.5. Nutrient Intake

There was an effect of the interaction between the use of PE and the climatization regimen only for water intake and a trend toward an interaction for NDF consumption (Table 7). There were significant reductions in the intake (kg/d) of OM (23.4 vs. 22.8), starch (7.68 vs. 7.47), EE (0.85 vs. 0.82), and ADF (4.37 vs. 4.23) based on the mean values for R10 and the mean values for R5. The intake of ash and CP tended to decrease with the use of R10. The consumption of NDF was higher for CTR5 than for CTR10 and PER10 but did not differ from PER5. The use of PE significantly reduced water intake when the largest number of cooling cycles was used (R5); however, the intake was not different between the spray cycles with the CT treatment. For the particle selection index, there was no significant difference among treatments.

### 3.6. Serum Parameters

AST enzyme activity was not influenced by any of the treatments, and there was no significant difference or interaction between treatments (Table 8). A trend toward an interaction was observed for GGT activity, such that the use of the R10 regimen increased GGT activity when PE was used, differing only when compared to that for CTR10. Total protein and albumin values were influenced by the acclimatization regime, with an increase for R10 (6.77 vs. 7.16 and 3.06 vs. 3.30, respectively). Conversely, triglycerides tended to increase with the use of PE, regardless of the number of spraying cycles. However, glucose, globulin, and urea levels were not altered.

## 4. Discussion

### 4.1. Environmental Conditions

The environmental variables observed during the experimental period evidenced the thermal oscillation characteristics of the southern region of Brazil. The system, the constructive characteristics of the facility used, and the microclimatic characteristics of the experimental site contributed to the thermal conditions to which the animals were exposed throughout the experiment. The THI has been widely used to characterize the microclimate in studies conducted with dairy cattle in different countries [31,32] and adequately described the thermal conditions of the trial. According to the THI values, the experimental conditions of this trial could be classified as potential thermal discomfort for lactating cows because recent studies have shown thresholds of 68 for thermal discomfort [33] and 65 for animals with high productive potential [34]. In our study, as previously shown, the THI average was above 65.

### 4.2. Physiological Parameters

The environmental conditions experienced by the animals in our study led to a mitigating effect of thermal stress, both for R5 and R10, with an interaction with PE for TS. Throughout the day, there was a reduction in TS and RR, but at 8:00, RR and TS were greater than at other times, indicating that the activation parameters of the cooling system must be revised. If only air temperature is taken into account as a system activation criterion, the correct moment for system activation may be underestimated. The THI values were above 65 in the first hours of the day. Our findings confirm the thermal discomfort condition, as described by [34]. In addition, under heat stress conditions, physiological changes in animals are used to reestablish ideal body thermal conditions and decrease body temperature through the activation of physiological mechanisms that increase the RR and sweating, promoting the loss of heat through thermal exchange [35,36]. The effect of the interaction of PE significantly reduced ST in the PER5 treatment, with no significant difference in the other treatments. This direct relationship with RR was observed because in the same treatment, RR tended to decrease.

Further investigation of the response related to the effect of PE in mammals suggests that PE and its analogs exert their effects by binding and activating the transient receptor potential cation channel [37]. The reduction in ST is possibly related to this receptor being a nonselective cationic channel widely expressed in the body. It is present mainly in sensory neurons of various tissues of the peripheral region and in the brain, lungs, liver, spleen, intestines, kidneys, stomach, bladder, and reproductive tract but is also found in mucosal epithelial cells, the epidermis, the vascular endothelium, and some immune cells [38]. Our study used a low dosage, possibly imparting additional dose-dependent responses. As reported in the literature, linear effects as a function of dosage were observed [39,40,41,42,43].

The RR obtained was directly associated with ST. These values are considered mild heat stress levels, and ST has a high correlation with respiratory rate in cows [44]. These parameters are used to demonstrate the beneficial effects of intermittent cooling [14]. The effect of PE associated with lower cooling intermittency (R5) improved heat exchange with the environment, favoring the thermal equilibrium of the animals, as demonstrated by the lower ST. Data reported in the literature showed that direct evaporative cooling affects the thermoregulatory metabolism of animals, supporting the cow’s thermal comfort [45,46,47]. Despite these thermoregulation benefits by PE, especially when associated with the R5 regime, milk production and DM intake were reduced compared to those for CT.

To effectively control homeostatic mechanisms, RT is a simple and efficient means to control the internal temperature of animals. In this study, the RT averaged 38.4 °C, which is considered physiologically normal. In addition, RT did not differ among treatments and was even below the average of 38.7 °C observed in an optimized cooling system [15]. In our study, the RT indicated that the thermoregulatory mechanisms of the animals were sufficient to maintain thermal equilibrium under the experimental conditions; however, the R10 sprinkler regimen required greater physiological effort by cows to obtain this balance.

In a study with rats, ref. [48] reported that when 5 mg/kg capsaicin was provided, body temperature decreased (37.1 vs. 36.8 °C) compared to that for rats that did not receive capsaicin. These results indicate the possibility that capsaicin activates heat-sensitive neurons and inhibits cold-sensitive neurons via TRPV-1 in the preoptic area of the hypothalamus, acting directly on heat-sensitive nerve terminals [13,49]. In general, the excitation of these heat-sensitive nerve cells activates heat loss mechanisms [48]. Thus, in terms of reductions in ST and RR, these responses of heat-sensitive neurons might explain the heat loss induced by capsaicin.

### 4.3. Behavior

It is possible through behavioral assessments to define strategies that optimize the use of resources in animal production. There was an interaction effect between the sprinkler regimen and the use of PE for time spent eating each visit. The spraying regimen was evaluated with less intermittence, and the greater number of spraying cycles (R5) increased the time of permanence in the feeding area when no PE was used. Thus, the CTR5 treatment resulted in the longest time spent at each visit and possibly a larger meal size, which explains the higher nutrient intake previously discussed. However, the use of PE did not affect the time spent at each visit.

Regarding ingestive behavior, the time that the animals remained eating was longer when the cows were fed PE. This observation was confirmed when we verified the intake rate, as animals that received PE ate on average 64 g DM/minute, while the CT group ate 80 g DM/minute: 25% more without the use of additives. The literature describes that the use of essential oils in the diet of production animals promotes better palatability and increased DM intake [50,51]. We did not find an isolated effect for the different intermittences in relation to feeding time.

The CT treatment increased the time spent lying down on the padded area by 10.6% compared with the PE treatment (on average 12.5 h/day vs. 11.2 h/day, i.e., 1 h and 18 min more per day). For the same parameter (lying time) and the R5 regimen, the animals spent 48% ± 2 of the time lying down [15]. The time spent lying down by dairy cows has a direct impact on productive responses, comfort, and well-being. This behavior may favor the better performance obtained by CTR5 treatment because cows that remain standing longer have limited blood flow to the udder, the amount of nutrients available to the mammary gland is lower, and nutrient absorption is reduced, compromising milk production [6]. Information on the effects of PE on the behavior of cattle is scarce in the literature, and we do not have information on its effects.

The presence in the feeding track directly correlated with feeding time and time in the resting area, and this result highlights the effects of the additive on the imposed treatments. Based on the results, there was an effect of using water in the cooling system, which was offered continuously to the animals whenever the temperature was above 22.2 °C. Animals that received the CT treatment remained in the cooling area 25% of the time, while animals that received PE remained in the cooling area 31.6% of the time, a 22% increase in the use of cooling, data that can guide the optimization of water and electricity use.

Future studies should review the dosage of PE, as recent studies have shown the possibility of increasing results using higher dosages [52]. The optimization of water resources in production systems is a need that can contribute to sustainability and the conscious use of water [11]. Currently, production costs are high, creating challenges regarding the financial management of properties, and water availability is decreasing. Future research should consider the use of new technologies that contribute to the efficient management of water resources, which are essential to milk production systems.

### 4.4. DM Intake and Milk Production and Composition

Our experiment was developed in an experimental shed under management conditions adopted by commercial properties, with the animals allowed free access to the feeding and cooling system. Under these conditions, we observed that the exposure of the animals to cooling with more spraying cycles (R5) increased DM intake and milk production. Cows that received the CTR5 treatment had greater DM intake than did those that received other treatments, producing 3.1, 3.3, and 3.8 kg/day more than PER10, CAPR5, and CTR10, respectively. The shortest intermittence time (interruption of sprinkling) and therefore the highest number of sprinkler cycles increased milk production by 3.8 kg/day (10.7% increase). However, an additional 4.8 L/cow/h of water was used (considering 2.5 cows/sprinkler and a mean daily time of 8.9 h/day for the activated system), corresponding to an additional 43 L of water per cow per day.

The results obtained in [11], in which two spray water flow coefficients were compared, indicated that 1.3 L/min was as efficient as 4.9 L/min; however, these authors used 12 min cycles (3 min on and 9 min off). Our study instead used a fixed flow coefficient of 4 L/min, varying the duration of the spraying cycles, a condition that was sufficient to demonstrate important performance effects.

In the R10 cooling system, the animals reduced their DM intake to reduce the adverse effects of heat, and consequently, lower nutrient volumes were available for milk synthesis, which was reflected in the production efficiency. Similar findings were reported by authors when comparing the DM intake, productivity, and feed efficiency of cows under thermoneutral conditions vs. heat stress conditions [53,54]. Thus, there was mitigation of the adverse effects of high environmental temperatures provided by lower intermittent cooling (R5) and therefore a greater number of spray cycles. Direct cooling through water spray, applied in more frequent cycles, improved thermal comfort because the water spray acted directly on the thermoregulatory mechanisms of the animals [14,54].

Changes in management practices, dietary adjustments, the use of additives, and innovative cooling strategies are alternatives to maximize productive and reproductive results and address the current need for water efficiency [6,11,14,15].

In our study, we included PE in the diet of the animals because its positive effects have been scientifically demonstrated. There was an interaction between the climate control regimen and the use of PE for milk production and ECM. Despite the higher milk production for CTR5 compared to the other treatments, when the production took into account the energy required for the synthesis of milk components (ECM), there was no difference between CTR5 and the treatments that used capsaicin (PER5 and PER10). PE has been studied in dairy cattle, but until now, there has been no investigation of combining feed additives with a microclimate control system. Changes in feed intake and enzyme secretion as well as physiological effects have been described [43] with capsaicin. The feed efficiency results for CTR5 were greater than those for CTR10 and PER5 but did not differ from those for PER10.

From a comparison of our results with those in the literature, we assume that the effects of capsaicin in this study were not apparent because of the lower dosage used. In our study, the animals were supplemented with 40 mg/d capsaicin. Some studies reported no observed effect on milk production [55,56], but other studies have reported beneficial effects of capsaicin supplementation [40,41,57]. Dietary capsaicin supplementation had no effect on productivity in dairy cows [55], and an abomasal infusion (2 g/d) of PE (6% capsaicin) decreased milk production in dairy cows by 2.2 kg/d [57]. However, ref. [40] reported that supplementation with doses of 53 to 106 mg of capsaicin tended to increase milk production and increased fat-corrected milk production by up to 6.2. The authors in [41] reported that rumen-protected capsaicin supplementation (100 or 200 mg/d) increased milk production by 6.8%, while [42] reported a 9% increase in milk production by cows supplemented with 100 mg/d of rumen-protected capsaicin. When feeding cows rumen-protected capsaicin (100 or 200 mg/d), ref. [57] observed that supplementation increased feeding efficiency in dairy cows.

The protein content of milk tended to decrease with the effect of CTR5, which can be explained as an effect of dilution due to the higher production obtained. In this case, it is suggested that the higher DM intake promotes a greater amount of nutrients that can be synthesized, potentially promoting greater microbial protein synthesis and favoring milk protein yield. The results were similar for lactose: the daily yield was higher for CTR5 than for the other treatments. With regard to lactose, the greater supply of glucose originating from propionate favors greater milk production, which is closely linked to the synthesis of lactose [58]. An investigation of the effects of capsaicin on milk composition [59] reported no effects on milk fat and lactose content, with a trend toward increasing the protein content with respect to the protein content generated with the control treatment. These same researchers noted that there is no clear hypothesis to justify this trend because in their study, rumen degradation of the protein, its digestibility in the total tract, and the proportions of propionate in the rumen were not affected by PE.

Milk quality, as measured by the SCC index, was not affected by treatment effects, corroborating the same conclusions of previous studies conducted by [40,43].

### 4.5. Nutrient Intake

There are many strategies to mitigate the effects of heat stress, including dietary adjustments, such as increased dietary energy concentration and decreased forage percentage, aiming to avoid a decrease in DM intake and promote greater nutrient intake [60]. In their study, ref. [61] evaluated the physiological effects and ingestive behavior of cows exposed or not exposed to evaporative cooling. The authors suggested that some concepts should be reviewed and that further research should investigate ingestive behavior because in their results, animals under acute thermal stress selected in favor of larger particles than did the group under the effect of evaporative cooling. Conversely, in our study, there was no difference in the particle selection index, indicating that the environments provided by the two cooling regimens evaluated were not acute thermal stress conditions, which could cause changes in these parameters. In addition, the processing of roughage and TMR consistency provided during our study may have minimized the selective effects of the animals [19].

The higher nutrient intake observed in animals that received R5 cooling is directly associated with higher DM intake compared to the other treatments. When the effect of PE was analyzed, there were no significant effects on nutrient intake and particle selection. In the literature, there are no reports of PE’s effects on these cited variables, which was one of the motivations for our study.

There was an interaction between the use of PE and the cooling regimen for water consumption. Greater water consumption was observed by R10, although the R5 system required greater water use for direct cooling. The water consumption was 26 L/day less for the animals in the PER5 treatment. In [62], the authors reported that beef heifers supplemented with capsaicin (1 g/d) exhibited an almost 26% increase in water intake. Similarly, ref. [63] reported a decrease in DM intake during the first 2 h after feeding for heifers supplemented with capsaicin (0.125, 0.25 or 0.5 g/d), but there was a linear increase in total DM intake when compared to that for the control and a strong correlation between water intake and DM intake (r = 0.98). In both studies, the additive was offered once a day mixed with a small amount of concentrate to ensure consumption. The authors suggested that the observed increase in feed intake is possibly related to the pungency of capsaicin and the increase in water intake.

Water consumption is also related to environmental factors that directly influence THI. When animals experience THI > 68, they change their behavior, prioritizing areas close to a drinking trough due to the proximal microclimate and increasing water consumption to maintain an osmotic balance in cells [64]. This is the first study to investigate the effect of intermittent evaporative cooling systems and capsaicin supplementation, as there are no reports in the literature related to capsaicin supplementation and water consumption; however, evidence indicates a relationship between capsaicin and the intermittent systems used. The greater water consumption compensates for the losses of liquids by panting and sweating and favors heat loss [65]. The results illustrate a positive effect, attenuating the adverse thermal conditions. The authors in [15] emphasized the importance of strategies adopted for the better use of water resources, enhancing their effect, in combination with dietary inclusions that favor animal performance [66].

### 4.6. Serum Parameters

The AST and GGT values indicated normal liver activity for high-production Holstein cows, with reference values ranging from 58 to 100 and 22 to 64 IU/L, respectively [67]. The GGT enzyme serves as a transport molecule and helps the liver metabolize drugs and other toxins [68]. Increased serum GGT levels indicate liver damage [69,70,71]. These results are similar to the findings by [72] and those of our study. The authors in [45] studied PE supplementation in lactating cows during the summer and concluded that PE had an effect on different body functions without affecting liver function and even improved the metabolic activity of cows during heat exposure. The results obtained for these enzymes indicate that PE had no negative effect on the liver activity of lactating cows; ref. [73] studied the effects of capsaicin on liver activity in rats and reported hepatoprotective and antioxidant effects.

In our findings, we observed only a slight difference in serum metabolism only for total protein and albumin, differing only for the different cooling systems. Albumin is produced by the liver, and its increased synthesis may indicate good liver health, which may be an indirect cooling response that allows the liver to be more efficient due to the general physiological conditions of the animal because the liver is compromised under heat stress conditions [74].

## 5. Conclusions

The interaction between the cooling regimens evaluated and the use of PE was not very evident, and the factors mostly interfered independently with the studied variables. However, the surface temperature of the animals, which is an important indicator of heat stress, was affected differently by the cooling regimens, depending on the use or absence of PE. Pepper extract potentiated the heat reduction in cows when they were subjected to the cooling regimen with a greater number of spray cycles. The cooling strategies were effective, but the regimen with the highest number of spray cycles improved milk efficiency and production.

## Figures and Tables

**Table 1:** Ingredients and chemical composition of the total mixed diet.

Ingredients %	(DM Basis)
Corn silage	46.1
Tifton hay	1.8
Concentrate	44.1
Wet citrus pulp	8.0
**Chemical composition**	
DM, %	46.0
Crude protein % DM	16.4
Neutral detergent fiber, % DM	27.7
Acid detergent fiber, % DM	17.6
Nonfiber carbohydrate, %DM *	46.5
Starch, % DM	30.5
NEl (Mcal/kg) *	1.53

* Estimated by [17].

**Table 2:** Microclimate variable (mean ± standard deviation) and range (min—max) by treatment.

Microclimate Variable	CT		PE	
	R5	R10	R5	R10
Air temperature (°C)	21.72 ± 2.99	21.78 ± 2.89	21.71 ± 3.14	21.80 ± 2.91
Temperature range (°C)	(15.25–32.02)	(15.30–31.94)	(15.20–32.51)	(15.34–31.94)
Relative humidity (%)	(81.73 ± 12.36)	(80.92 ± 11.76)	81.18 ± 12.54	80.42 ± 11.57
Humidity range (%)	(34.92–97.95)	(35.10–97.69)	(36.18–97.69)	(35.97–93.92)
THI	(69.80 ± 3.35)	(69.83 ± 3.21)	69.74 ± 3.53	69.81 ± 3.27
THI range	(61.43–80.42)	(62.09–80.08)	(61.52–80.89)	(61.53–80.26)

CT = Control treatment; PE = pepper extract; R5 = sprinkler cycles of 30 s on and 4.5 min off; R10 = sprinkler cycles of 30 s on and 9.5 min off (R10). The data are shown as the means ± standard deviation.

**Table 3:** Effects of different evaporative cooling regimens and the use or absence of PE on the respiratory rate (RR), surface temperature (ST), and rectal temperature (RT) of lactating Holstein cows (*n* = 8).

Item	CT		PE		Time				*p* Value			
	R5	R10	R5	R10	08:00	14:00	20:00	SEM	Time	Adit	Cool	Adit × Cool
RR mov./min.	59.0	61.0	54.2	58.3	62.3	58.9	53.3	0.77	<0.0001	0.0748	0.1410	0.5942
ST, °C	32.8 a	32.9 a	30.4 b	32.1 a	34.2	30.9	31.0	0.19	<0.0001	<0.0001	0.0028	0.0076
RT, °C	38.4	38.6	38.4	38.3	38.3	38.5	38.5	0.06	0.3171	0.1635	0.6825	0.3334

CT = Control treatment; PE = pepper extract; R5 = sprinkler cycles of 30 s on and 4.5 min off; R10 = sprinkler cycles of 30 s on and 9.5 min off (R10); SEM = standard error of mean; Adit = use of PE as additive; Cool = evaporative cooling regimens; Adit × Clim = interaction between cooling regimens and PE.

**Table 4:** Effects of different evaporative cooling regimens and the use or absence of PE on the feed behavior of lactating Holstein cows (*n* = 8).

	CT		PE			*p* Value		
	R5	R10	R5	R10	SEM	Adit	Cool	Adit × Cool
Eating								
min/visit	25.7 ^a^	19.0 ^b^	21.6 ^a,b^	22.3 ^a,b^	0.75	0.8028	0.0449	0.0165
min/day	703	615	805	682	34.0	0.2280	0.1356	0.7900
Visits, n^o^/day *	27.5	32.7	36.8	32.0	2.32	0.3773	0.9617	0.3111
Latency, min **	15.6	13.2	13.8	21.0	1.58	0.3414	0.4437	0.1349

CT = control treatment; PE = pepper extract; R5 = sprinkler cycles of 30 s on and 4.5 min off; R10 = sprinkler cycles of 30 s on and 9.5 min off (R10); SEM = standard error of mean; Adit = use of PE as additive; Cool = evaporative cooling regimens; Adit × Clim = interaction between cooling regimens and PE; * counted as a visit only when cow spent more than 30 s on the feed bunk; ** latency represents the time (minutes) between PE consumption and drinking water. Means followed by different letters in the line are different at the 5% level.

**Table 5:** Effects of different cooling regimens and the use or absence of PE on the behavior of lactating Holstein cows (*n* = 8).

Behavior (min/h)	CT		PE			*p* Value		
	R5	R10	R5	R10	SEM	Adit	Cool	Adit × Cool
Standing	24.0	23.7	28.0	26.9	0.80	0.0666	0.7072	0.8271
Lying down	31.5	31.3	27.5	28.6	0.94	0.0837	0.8206	0.7474
Idle	10.3	13.3	9.85	9.63	0.88	0.1689	0.3350	0.2666
Ruminating, min/day *	542.2	541.4	500.3	535.2	6.03	0.1888	0.3412	0.3223
Walking	2.48	3.94	4.09	3.36	0.32	0.4294	0.5781	0.1010
Eating	13.9	11.9	16.5	16.3	0.68	0.0375	0.5118	0.5854
Drinking	2.88	2.96	1.90	2.56	0.67	0.2831	0.5608	0.6466
Cows location								
At the feed bunk	15.5	14.6	20.2	17.9	0.65	0.0134	0.2921	0.6465
At the bed	40.0	40.4	34.7	36.7	1.06	0.0192	0.5090	0.6686

CT = control treatment; PE = pepper extract; R5 = sprinkler cycles of 30 s on and 4.5 min off; R10 = sprinkler cycles of 30 s on and 9.5 min off (R10); SEM = standard error of mean; Adit = use of PE as additive; Cool = evaporative cooling regimens; Adit × Clim = interaction between cooling regimens and PE; * counted as a visit only when the cow spent more than 30 s on the feed bunk; * daily mean time recorded by collar.

**Table 6:** Effects of different evaporative cooling regimens and the use or absence of PE on milk yield and milk composition of lactating Holstein cows (*n* = 8).

Variables	CT		PE			*p* Value		
	R5	R10	R5	R10	SEM	Adit	Cool	Adit × Cool
Dry matter intake	25.4	24.5	25.0	24.6	0.21	0.6642	0.0205	0.4222
Milk yield, kg/d								
Actual	39.3 ^a^	35.5 ^b^	36.1 ^b^	36.3 ^b^	0.50	0.0014	<0.0001	<0.0001
4% FCM ^1^	36.7	32.2	34.0	33.7	0.77	0.5898	0.0359	0.0627
ECM ^2^	36.8 ^a^	32.4 ^b^	33.9 ^a,b^	33.8 ^a,b^	0.70	0.4251	0.0180	0.0239
Milk fat								
%	3.67	3.81	3.97	3.45	0.11	0.8914	0.3753	0.1389
kg/d	1.40	1.23	1.33	1.25	0.04	0.7003	0.0896	0.6038
Milk protein								
%	3.45 ^b^	3.54 ^a^	3.54 ^a^	3.48 ^b^	0.03	0.4643	0.7059	0.0028
kg/d	1.34 ^a^	1.20 ^b^	1.21 ^b^	1.22 ^b^	0.02	0.0176	0.0022	0.0011
Milk lactose								
%	4.52	4.50	4.49	4.55	0.02	0.7836	0.3753	0.0917
kg/d	1.76 ^a^	1.53 ^b^	1.57 ^b^	1.61 ^b^	0.04	0.0501	0.0022	<0.0001
Milk efficiency								
Actual/DM intake	1.55 ^a^	1.46 ^b^	1.47 ^b^	1.49 ^b^	0.50	0.1794	0.0525	0.0056
FCM/DM intake	1.47	1.32	1.43	1.36	0.03	0.9923	0.0447	0.4690
ECM/DM intake	1.47	1.33	1.43	1.36	0.03	0.9054	0.0323	0.4217
CCS × 10^3^	2.42	2.48	2.41	3.06	0.25	0.4545	0.3656	0.4364

CT = control treatment; PE = pepper extract; R5 = sprinkler cycles of 30 s on and 4.5 min off; R10 = sprinkler cycles of 30 s on and 9.5 min off (R10); SEM = standard error of mean; Adit = use of PE as additive; Cool = evaporative cooling regimens; Adit × Clim = interaction between cooling regimens and PE. ^a,b^ Means followed by different letters in the line are different at the 5% level. ^1^ FCM was calculated as milk yield (kg/d) × [milk fat (%) × 0.15 + 0.4]. ^2^ ECM was calculated as milk yield (kg/d) × {[0.3887 × milk fat (%)] + [0.2356 × milk protein (%)] + [0.1653 × milk lactose (%)]}/3.1338 [26].

**Table 7:** Effects of different evaporative cooling regimens and the use or absence of PE on nutrient intake, water consumption, and the sorting index of lactating Holstein cows (*n* = 8).

Variables	CT		PE			*p* Value		
	R5	R10	R5	R10	SEM	Adit	Cool	Adit × Cool
Intake kg/d								
OM	23.6	22.8	23.3	22.9	0.20	0.6739	0.0187	0.4534
Ash	1.81	1.74	1.77	1.76	0.01	0.5631	0.0630	0.1496
CP	3.84	3.79	3.87	3.76	0.04	0.9787	0.0615	0.4131
Starch	7.73	7.44	7.62	7.49	0.07	0.7030	0.0169	0.3789
EE	0.85	0.82	0.84	0.82	0.01	0.8144	0.0254	0.7371
NDF	8.09	7.72	7.76	7.73	0.05	0.0866	0.0023	0.0582
ADF	4.33	4.25	4.41	4.20	0.04	0.7806	0.0119	0.2450
Water consumption, L/d	82.5 ^b^	87.7 ^a,b^	67.9 ^c^	94.3 ^a^	1.66	0.0725	<0.0001	<0.0001
Sorting index	100.8	100.5	101.3	100.7	0.17	0.1962	0.1194	0.6010

CT = control treatment; PE = pepper extract; R5 = sprinkler cycles of 30 s on and 4.5 min off; R10 = sprinkler cycles of 30 s on and 9.5 min off (R10); SEM = standard error of mean; Adit = use of PE as additive; Cool = evaporative cooling regimens; Adit × Cool = interaction between cooling regimens and PE. ^a,b,c^ Means followed by different letters in the same row are different at the 5% level.

**Table 8:** Effects of different evaporative cooling regimens and the use or absence of PE on the enzymatic and metabolic status of lactating Holstein cows (*n* = 8).

Variables	CT		PE			*p* Value		
	R5	R10	R5	R10	SEM	Adit	Cool	Adit × Cool
Enzymatic								
AST (U/L)	94.4	85.6	92.7	84.5	4.21	0.7946	0.1182	0.9589
Gama GT (U/L)	50.4	48.4	50.9	56.3	1.93	0.0328	0.3607	0.0581
Metabolism								
Glucose (mg/dL)	51.1	51.4	53.4	50.4	1.26	0.7191	0.5601	0.4295
Total protein (g/dL)	6.78	7.15	6.76	7.16	0.07	0.9770	0.0038	0.9266
Albumin (g/dL)	3.15	3.36	2.98	3.25	0.07	0.2269	0.0336	0.7844
Globulins (g/dL)	3.66	3.49	3.71	3.80	0.11	0.3061	0.8080	0.4410
Triglycerides (mg/dL)	25.1	25.4	26.2	27.3	0.47	0.0569	0.3616	0.6213
Urea (mg/dL)	45.6	46.7	40.9	46.1	1.49	0.3335	0.2517	0.3719

CT = control treatment; PE = pepper extract; R5 = sprinkler cycles of 30 s on and 4.5 min off; R10 = sprinkler cycles of 30 s on and 9.5 min off (R10); SEM = standard error of mean; Adit = use of PE as additive; Cool = cooling regimens; Adit × Cool = interaction between cooling regimens and PE.

## Data Availability

Not applicable.
